# Community-based health and social care demand associated with frailty in people aged 50 and over in England 2025–40: routine data analyses and simulation modelling

**DOI:** 10.1093/ageing/afag256

**Published:** 2026-08-23

**Authors:** Bronagh Walsh, Carole Fogg, Tracey England, Sally Brailsford, Martin J Vernon, Peter Griffiths, Lee-Ann Fenge, Gulam Muktadir, Evgeny Galimov, Abigail Barkham, Lyndsey Williams

**Affiliations:** University of Southampton - School of Health Sciences, Southampton, United Kingdom of Great Britain and Northern Ireland; University of Southampton - School of Health Sciences, Southampton, United Kingdom of Great Britain and Northern Ireland; University of Southampton - School of Health Sciences, Southampton, United Kingdom of Great Britain and Northern Ireland; University of Southampton - Southampton Business School, Southampton, Hampshire, United Kingdom of Great Britain and Northern Ireland; The ChristieNHS Foundation Trust, Manchester, England, United Kingdom of Great Britain and Northern Ireland; University of Southampton - School of Health Sciences, Southampton, United Kingdom of Great Britain and Northern Ireland; NIHR Applied Research Collaboration, Wessex; Bournemouth University Faculty of Health, Environment and Medical Sciences, Bournemouth, England, United Kingdom of Great Britain and Northern Ireland; Imperial College Health Partners, London, United Kingdom of Great Britain and Northern Ireland; Imperial College Health Partners, London, United Kingdom of Great Britain and Northern Ireland; Hampshire and Isle of Wight Healthcare NHS Foundation Trust, Southampton, United Kingdom of Great Britain and Northern Ireland; NHS North West London ICB, London, United Kingdom of Great Britain and Northern Ireland

**Keywords:** frailty, mental health services, community health services, social care, system, older people

## Abstract

**Background:**

In ageing populations, frailty prevalence and associated demand for health and social care services are expected to increase. Whilst there is good evidence about the demand for primary and secondary care services, the estimated demand for community-based health and social care associated with frailty is not known.

**Aim:**

To estimate future demand for community-based health services and publicly funded social care for those aged ≥50 with frailty, in England.

**Methods:**

Analysis of cohort data from North-West London provided rates of mental health, community health and publicly funded social care contacts among the population aged 50 and over, by age and electronic frailty index (eFI) categories. Using System Dynamics (SD) simulation modelling, mean service use and population estimates informed projections of national demand over 16 years (2025–40).

**Results:**

Over the simulation period, mental health, community health and publicly funded social care use are projected to increase by 16%, 30% and 30% respectively. Against baseline, lowering frailty incidence could reduce mental health, community health and local authority social care contacts by 1.4, 10 and 400 000 m respectively, between 2025 and 2040. Slowing frailty progression could reduce mental health, community health and social care contacts, compared with baseline, by 2.8, 45.3 and 2 m respectively over the same period.

**Conclusions:**

Demand for community-based health and social care services will increase substantially as the population ages and becomes more frail. Reduced frailty incidence and progression could slow demand growth, but demand will continue to grow due to large numbers already living with mild to moderate frailty.

## Key Points

- Community-based health and social care use associated with frailty will increase in future.
- Reducing frailty incidence and progression could reduce community service demand against baseline.
- Reductions in demand from lower incidence or progression are offset by increased demand due to population ageing.

## Background

The UK population is ageing, with projected increases in those aged 50+ from 22 million in 2022 to 26.3 million in 2044 [1–3]. Ageing is associated with frailty, a clinical state of compromised ability to cope with stressors, resulting from age-associated declines in physiological reserve and function [4]. It is estimated that 1.8 million people in the UK aged ≥60 were living with frailty in 2016 [5] and prevalence increased from 27% (2006) to 39% (2017) in England, with 11% of those aged 50–64 already living with frailty [6]. Prevalence of mild to severe frailty (measured via the eFI) in those aged 50 and over is projected to rise further, to 76% in 2040 [3]. Primary and secondary care service use and associated costs are higher in adults living with frailty and increase with the severity of frailty [7, 8]. Projected growth in frailty will be associated with increased service use costs in these services of £10 billion by 2040 [2, 3] and numbers of people with complex care needs are predicted to increase substantially by 2035 [9]. There is, however, evidence of unmet need for health and social care, with significant health inequalities related to deprivation [10, 11] and this gap between need and capacity will continue to expand. In this context, robust service and workforce planning to meet future health and social care needs of those living with frailty is essential to better match service demand and workforce capacity to meet the needs of the ageing population [12–15].

Providing care for older people with frailty is complex, requiring an integrated approach from primary, secondary, community and social services [16, 17]. Although there is a growing body of evidence on primary and secondary health care utilisation in older people with frailty, less is known about their community-based health, mental health and publicly funded social care use. There is some evidence that increased frailty in ageing populations will result in increased demand for social care in those aged 85 and over [18, 19]. Although it is recognised that new ways of working will be needed to address demand for community-based services, particularly in deprived areas [20, 21], the shift to home-based care will require better understanding of trends in demand for community-based care and prevention and early intervention services [22]. This study aimed to address the evidence gap around the impact of frailty on community-based health and social care for people aged 50 and over, to provide projections of demand as the population ages and to inform service and workforce planning.

### Aim

To fill the knowledge gap around community-based services in older people with frailty, by estimating future demand for community health, mental health and publicly funded social care for those aged ≥50 with frailty, in England, through the use of a simulation model informed by linked routine data analysis.

### Methods

#### Study design

Retrospective analysis of electronic health records, combined with System Dynamics (SD) simulation modelling. SD modelling has often been used to model healthcare systems to provide a strategic view of a system [23–26]. It is ideally suited to model large populations where individual variability is not a major consideration.

#### Data source

The Discover-NOW Research Environment hosts a de-identified dataset linking depersonalised, contemporary, primary, acute, community and mental healthcare and social care electronic patient records from over 2.8 million patients in North-West London (NWL) registered at 344 GP practices [27, 28]. The Discover-NOW dataset is one of Europe’s largest linked longitudinal datasets capturing a population of around a third of London, an ethnically highly diverse population. The number of participating GP practices remained stable during the data collection period, varying between 351 and 357.

#### Participants

Patients aged 50 and over registered at NWL General Practitioner (GP) practices contributing to the Discover-NOW databank between 2015 and 2022 were eligible. This age range allowed consistency with previous simulation modelling [3], and service use to be tracked in frailty in the ageing population; previous analyses demonstrated that frailty is already present over 10% of those aged 50–64. An open cohort design enabled the addition of patients who turned 50 or moved to a participating practice and were present on 1 January of a calendar year in the study period. Patients left the cohort by leaving the participating practice or dying.

Age was categorised into four groups, reflecting groupings reported in literature relating to older adults’ healthcare, and cut-offs for services recommended by the Stakeholder Engagement Group (SEG) in a linked study: 50–64, 65–74, 75–84 and ≥85 [2, 29]. Frailty was categorised using electronic Frailty Index (eFI) score, a ‘cumulative deficit’ index, measuring frailty through accumulation of a range of deficits [30]. The eFI score indicates the number of deficits present out of a possible total of 36, with higher scores indicating more severe frailty [30]. An eFI score was calculated on 1 January for each patient and the relevant frailty category assigned (fit, mild, moderate, severe).

#### Service use data

All mental health, community health trusts and local authority social care services in NWL contributed data. Mental health contacts included psychiatry, older persons mental health services, crisis resolution and liaison teams, with a contact representing a face-to-face or telephone appointment, day case or inpatient admission. Mental health service use, although not directly attributable to frailty, was included following consultation with the SEG due to prevalence of anxiety and depression in older people and associated service use [31] [32, 33]. Community health contacts covered physiotherapy, telehealth, rehabilitation and community nursing. For social care, a contact was defined as either an assessment, new care package or a change in care package, and included services such as personal home care, domestic support, home adaptations and respite care. Service use contacts for each service type reflect heterogenous administrative records, collated to capture broad activity within different services; they do not reflect time or intensity of input. Social care assessments are important to service provision in frailty, and have therefore been included, though they may not always lead to provision of a care package. Service use data were used to generate average yearly service use rates, which were applied to all simulation years.

#### Analysis

For each of the services (mental health, community health, publicly funded social care), the proportion of follow-up years with a contact was calculated for each of the 16 age and frailty combination categories. For each category, the average (standard deviation) number of contacts per year was calculated for patients with at least one contact in a year.

#### Simulation model development

The Frailty Dynamics Model provided population-level estimates of frailty incidence, prevalence and rates of frailty transitions (adjusted for deprivation, sex, ethnicity and urban location) for England [29, 34]. Estimated transitions from fit to any level of frailty were 48/1000 person-years aged 50–64, 130/1000 person-years aged 65–74, 214/1000 person-years aged 75–84 and 380/1000 person-years aged ≥85 [2]. This model was developed to provide projections of frailty prevalence in the English population [3]. The population structure within the model aged ≥50 is considered as 16 separate subgroups, categorised according to age group (50–64, 65–74, 75–84 and 85+) and frailty category (fit, mild, moderate and severe) [29].

Discover-NOW data analyses provided average community health and social care service use rates for each of the 16 age/frailty groups over the study period (2015–22). Average rates were applied to the national-level estimates of people within each frailty category to provide projections of the number of contacts in each of the three services in England during 2025–40. The service use data were derived from an area with higher deprivation and diversity than the average for England; applying national frailty transition rates and service use rates per age and frailty sub-group reduced the risk of over-estimating service use for the whole population. The simulation assumes that service use rates are constant for each of the age/frailty subgroups throughout the simulation.

#### Validation of the simulation model

The simulation model projections were internally validated against service use data from Discover-NOW and externally validated against available NHS Digital data for the development period to the present. Comparison between the mental health projections and national dashboard figures was close (See Appendix 1 in the Supplementary Data Section for details). The comparison for community health was less close, but national data were more complete in later years for which the model provides much closer estimates (See Appendix 2 in the Supplementary Data Section for details). Confidence in the future projections is therefore high for community and mental health care, and moderate for social care, for which the simulation model was closer to national dashboard data for all adults (See Appendix 3 in the Supplementary Data Section for details).

#### Model estimation and scenario testing

Projected service use contacts for the simulation period (16 years inclusive) for each frailty category are presented in Table 3. Two illustrative ‘what-if’ scenarios are considered alongside the baseline (no change to services or frailty trends) experiment to estimate impact of broad policy and practice shifts. The scenarios were identified through consultation with the SEG [2] for their potential to be useful to commissioners and service planners. Following prioritisation of the scenario options, the parameters for the scenarios were informed by review of literature evidence as per a previous study [7]. The scenario experiments were as follows:

1. Reducing Incidence: Fit-to-Mild transition rates for all age groups were reduced by 5%, thus reducing incidence of frailty in the population, representing the potential impact of general public health measures or the introduction of services targeting those that are pre-frail or at risk of developing frailty. Whilst studies have suggested potential reductions of up to 20%, this was considered a more feasible reduction [2, 35–40].
2. Reducing progression: frailty progression slowed by reducing Mild-to-Moderate transition rates by 10% and Moderate-to-Severe by 5% in each age group [2, 35, 41, 42]. The aim was to consider the impact of clinical or public health intervention.

## Results

Patients aged 50 and over registered with GPs in the NWL area between 2015 and 2022 were analysed, including 480 663 patients in 2015 and 701 858 by 2022 (890 335 patients contributing overall). Over the study period, 409 672 patients entered (225 603 turning 50 and the remainder due to registrations at GP practices), 67 107 died (7.54%) and 147 728 (16.59%) de-registered. Tables 1 and 2 describe the number of patient-years with contact with mental health, community health or publicly funded social care services and the average use within a year for people in the 16 age/frailty categories. There were 121 334 patient-years with at least one contact with mental health services, with the average number of contacts in a year varying between 7.0 (Severe, 85+) and 12.9 (Fit, 50–64). For community health, there were 663 969 patient-years with contacts, with the average number of contacts varying between 5.2 (Fit, 50–64) and 25 (Severe, 85+). For social care, there were 68 677 patient-years, with the average number of contacts varying between 4.5 (Fit, 50–64) and 5.6 (Severe, 65–74).

**Table 1:** Proportion of years (2015–22), in NWL, with at least one contact with community health, mental health or social care services, age group and frailty severity.

	Frailty severity (eFI category)				
	Fit	Mild	Moderate	Severe	Overall
Person-years	*N* = 3 080 383	*N* = 1 058 114	*N* = 383 984	*N* = 183 572	4 706 503
***Age group***					
**Community health services**					
50–64 years	114 894, 5.1%^a^	80 371, 18.1%	28 524, 36.0%	9086, 57.5%	232 875, 8.4%
65–74 years	42 511, 6.9%	64 467, 19.0%	41 324, 36.8%	22 200, 59.5%	170 502, 15.4%
75–84 years	18 865, 9.8%	51 660, 23.3%	56 026, 41.4%	48 161, 63.3%	174 712, 27.9%
85+ years	4811, 14.7%	16 851, 31.2%	27 861, 49.0%	36 357, 66.9%	85 880, 43.4%
Overall	181 081, 5.9%	213 349, 20.2%	153 735, 40.0%	115 804, 63.1%	663 969, 14.1%
	
**Mental health services**					
50–64 years	27 058, 1.2%	19 200, 4.3%	7163, 9.0%	2293, 14.5%	55 714, 1.2%
65–74 years	5739, 0.9%	8614, 2.5%	6079, 5.4%	3821, 10.2%	24 253, 0.5%
75–84 years	3394, 1.8%	8062, 3.6%	8879, 6.6%	8342, 11.0%	28 677, 0.6%
85+ years	879, 2.7%	2366, 6.6%	4048, 7.1%	5397, 9.9%	12 690, 0.3%
Overall	37 070, 1.2%	38 242, 3.6%	26 169, 6.8%	19 853, 10.8%	121 334, 2.6%
					
**Social care services**					
50–64 years	3990, 0.18%	5063, 1.1%	3237, 4.1%	1598, 10.1%	13 888, 0.5%
65–74 years	2055, 0.33%	4149, 1.2%	4476, 4.0%	3999, 10.7%	14 679, 1.3%
75–84 years	1796, 0.93%	4907, 2.2%	7396, 5.5%	9649, 12.7%	23 748, 3.8%
85+ years	802, 2.5%	2273, 4.2%	4766, 8.4%	8521, 15.7%	16 362, 8.3%
Overall	8643, 0.28%	16 392, 1.5%	19 875, 5.2%	23 767, 12.9%	68 677, 1.5%

^a^Percentage denominators are the number of person-years for the corresponding age/frailty category (data not shown)

**Table 2:** Average number of contacts with community health, mental health and social care services in years with at least one patient contact in NWL (2015–22), age group and frailty severity.

	Frailty severity (eFI category)			
*Age group*	Fit	Mild	Moderate	Severe
	**Mean (SD)**	**Mean (SD)**	**Mean (SD)**	**Mean (SD)**
**Community health services**				
50–64 years	5.1^a^ (11.8)	7.5 (20.8)	12.7 (37.7)	22.4 (59.3)
65–74 years	6.6 (17.7)	8.5 (22.7)	13.8 (39.7)	23.4 (56.5)
75–84 years	10.1 (30.2)	11 (29.3)	14.9 (38.5)	24.5 (57.8)
85+ years	13.4 (32)	13.2 (30.6)	17.3 (38.7)	25 (52.8)

**Mental health services**				
50–64 years	12.9 (16.9)	12.4 (16.4)	12.6 (18.1)	11.5 (16.2)
65–74 years	11.7 (15.6)	10.7 (14)	10.1 (13.4)	9.9 (13
75–84 years	9.5 (11.2)	9.0 (11.1)	8.6 (10.4)	8.6 (11.05)
85+ years	8.1 (9.89)	7.3 (8.85)	7.1 (8.96)	7.0 (8.84)
				
**Social care services**				
50–64 years	4.5 (5.61)	5.1 (6.08)	4.99 (5.61)	5.5 (6.96)
65–74 years	4.8 (5.95)	4.95 (6.15)	5.2 (6.29)	5.6 (6.99)
75–84 years	4.8 (5.79)	5.1 (6.5)	5.1 (6.15)	5.3 (6.6)
85+ years	5.1 (6.13)	5.0 (6.15)	4.9 (5.93)	4.9 (5.9)

^a^mean (standard deviation) number of contacts in a year in which patients have at least one contact with the particular service sector

### Service use projections for England

Average service use rates for each age and frailty group were applied to national population projections for England, to derive annual service use projections for mental and community healthcare and social care in England for 2025–40 (Table 3). Estimates for the number of mental health contacts for those aged 50 and over increase from 10 475 794 to 12 162 939 (16.1%) across the observed period. The estimated number of community health contacts increases from 83 061 668 in 2025 to 108 139 309 (30%) in 2040. The estimated number of contacts with publicly funded social care increases from 3 637 987 in 2025 to 4 741 892 (30%) in 2040. In each of the services, the demand from fit and mildly frail patients decreases over time whilst the demand from those patients with moderate and severe frailty increases as more of the population become frail.

**Table 3:** Projected community health, mental health and social care service contacts in England for adults aged 50 and over, by frailty severity, 2025–40.

Year	Mental health services				Community health services				Social care services (local authority)			
*Frailty status*	*Fit*	*Mild*	*Moderate*	*Severe*	*Fit*	*Mild*	*Moderate*	*Severe*	*Fit*	*Mild*	*Moderate*	*Severe*
2025	1 012 097	3 761 942	3 334 964	2 366 790	2 706 212	16 298 992	27 611 575	36 444 890	103 572	686 908	1 239 912	1 607 595
2026	990 505	3 771 765	3 417 182	2 496 315	2 650 343	16 320 474	28 199 341	38 302 396	101 384	687 609	1 266 012	1 689 003
2027	968 480	3 772 026	3 490 076	2 622 498	2 594 247	16 310 632	28 724 976	40 107 418	99 091	686 726	1 289 068	1 768 001
2028	946 073	3 763 677	3 554 032	2 745 363	2 537 781	16 270 161	29 184 658	41 853 104	96 683	684 240	1 308 835	1 844 201
2029	924 011	3 748 244	3 608 952	2 862 431	2 485 894	16 224 426	29 611514	43 545 006	94 572	681 738	1 327 219	1 918 248
2030	905 226	3 728 803	3 655 653	2 972 857	2 444 467	16 185 878	30 015 796	45 181 386	92 975	679 825	1 344 803	1 990 134
2031	893 351	3 710 906	3 696 584	3 077 310	2 416 306	16 153 961	30 391 966	46 754 120	91 892	678 308	1 361 224	2 059 403
2032	885 375	3 695 583	3 733 185	3 177 128	2 393 326	16 115 239	30 722 426	48 252 231	90 928	676 381	1 375 484	2 125 343
2033	877 676	3 680 943	3 764 954	3 271 250	2 372 997	16 080 825	31 022 510	49 680 891	90 116	674 756	1 388 502	2 188 321
2034	870 254	3 667 189	3 791 223	3 354 686	2 359 557	16 081 903	31 348 670	51 055 144	89 918	675 548	1 403 564	2 249 908
2035	863 064	3 653 970	3 814 336	3 434 979	2 342 858	16 057 645	31 605 717	52 335 226	89 371	674 767	1 415 165	2 306 861
2036	855 745	3 642 379	3 835 597	3 512 329	2 322 535	16 015 703	31 808 006	53 530 056	88 581	672 979	1 424 105	2 359 717
2037	850 324	3 631 844	3 853 721	3 585 525	2 306 964	15 975 930	31 976 996	54 652 394	87 916	671 129	1431 417	2 409 165
2038	845 576	3 625 269	3 871 828	3 655 981	2 291 423	15 937 991	32 118 833	55 708 634	87 272	669 409	1 437 586	2 455 524
2039	840 741	3 622 273	3 890 385	3 723 922	2 274 242	15 901 472	32 239 957	56 705 620	86 609	667 874	1 442 978	2 499 168
2040	838 444	3 625 229	3 910 188	3 789 078	2 260 300	15 875 441	32 352 560	57 651 008	86 115	666 976	1 448 225	2 540 577

Differences in projected service use were explored under the two scenarios (Table 4). Under the first scenario (reduced incidence), the reduction in mental health contacts compared with baseline is between 67 510 and 95 160 each year, with a total of 1.4 million over the 16-year period. The reduction in community health contacts compared with baseline was between 426 329 and 809 942 each year, with a total of 10 850 449 over the 16-year period. The number of publicly funded social care contacts compared with baseline was reduced by between 18 299 and 34 609, with a total of 465 293 over the 16-year period. The reduction in number of mental health contacts could be doubled (to 2.8 million) if the second scenario (reducing frailty progression) is implemented. The reduction in number of community health contacts under the second scenario could be 45.3 million and the number of publicly funded social care contacts could be reduced by 2 million. Compared with the baseline scenario, reductions in service use are possible, but overall demand will continue to grow (See Appendices 4–6 in the Supplementary Data Section for further details).

**Table 4:** Differences in projected service use contacts for adults aged 50 and over across all frailty severities in England, between 2025 and 2040, for each scenario analysis.

	Reduced incidence			Reduced progression		
Year	Difference in mental health service use	Difference in community health service use	Difference in social care service use	Difference in mental health service use	Difference in community health service use	Difference in social care service use
**2025**	−67 510	−426 329	−18 299	−142 236	−2 112 574	−92 525
**2026**	−72 933	−475 904	−20 447	−152 521	−2 286 812	−100 028
**2027**	−77 626	−522 466	−22 460	−161 325	−2 441 158	−106 643
**2028**	−81 622	−565 633	−24 323	−168 775	−2 576 623	−112 417
**2029**	−84 933	−605 341	−26 029	−174 822	−2 693 943	−117 390
**2030**	−87 593	−641 489	−27 576	−179 511	−2 793 966	−121 603
**2031**	−89 727	−673 940	−28 958	−183 037	−2 877 658	−125 100
**2032**	−91 470	−702 620	−30 175	−185 655	−2 946 383	−127 942
**2033**	−92 842	−727 743	−31 236	−187 388	−3 001 419	−130 188
**2034**	−93 806	−749 427	−32 139	−188 005	−3 042 037	−131 805
**2035**	−94 487	−767 296	−32 882	−188 117	−3 071 369	−132 932
**2036**	−94 948	−781 620	−33 474	−187 836	−3 090 946	−133 638
**2037**	−95 125	−792 799	−33 930	−187 088	−3 102 328	−133 991
**2038**	−95 160	−801 121	−34 267	−186 065	−3 106 751	−134 050
**2039**	−95 082	−806 780	−34 491	−184 827	−3 105 185	−133 855
**2040**	−94 906	−809 942	−34 609	−183 385	−3 098 161	−133 429
**Total for all years**	−1 409 769	−10 850 449	−465 293	−2840 592	−45 347 312	−1 967536

## Discussion

This study provides novel analysis and projections of community-based service use for older people living with frailty, addressing an acknowledged evidence gap. Adding to evidence that frailty is associated with higher use of primary, secondary and urgent care services [7, 8, 43], this analysis provides new evidence about population demand for community-based health and social care services in older people living with frailty. [3, 7, 8, 44–48]. Service use increased overall with age and frailty, other than in mental health services, where use was highest in the 50–64 age group, suggesting need being met by other services in older age. Social care use was considerably lower than health service use, but data were only available for publicly funded care under current eligibility criteria; the demand for social care from all sources will be higher.

Model projections suggest that, over the next 16 years, the demand for community-based services will increase by up to 30% as frailty prevalence increases, consistent with previous simulation modelling for primary and secondary care [3]. The scenario experiments explored the impact on future demand if frailty incidence could be reduced or progression of frailty could be slowed, approaches in line with policy recommendations [49, 50] and NHS 10-year plan goals [21]. Scenario projections are illustrative for the purposes of comparison of different approaches, and absolute numbers should be considered in the context of underlying model assumptions, but the overall trends provide important messages for service development. Projected growth in community-based service use could be reduced, compared with baseline, by reducing frailty incidence or progression. However, in both scenarios, demand for care will continue to rise substantially even if these modest reductions are realised, highlighting the importance of service and workforce planning to meet the needs of the ageing population.

A recent audit of frailty identification and support provided in primary and community healthcare indicates there are still significant gaps in diagnosis and support, and therefore unmet needs among the population are likely to either persist or will lead to a steeper increase in caseloads without changes to service provision [51]. These model estimates may provide commissioners and planners with an insight into future demand for health and social care services to 2040. In addition, these analyses, coupled with caseload information, are being used to develop future workforce estimates for frailty [52].

### Limitations

These projections are in line with other analyses using eFI, but they should be considered in the context of other studies which include all frailty levels and use eFI [30] for frailty identification. As the eFI is a cumulative deficit index and the simulation model population includes only those aged 50 and over, the progression of frailty and increased prevalence reflect the ageing of this cohort and their accumulation of deficits. In addition, the eFI is generated from routine electronic health records, and so will necessarily reflect local differences in data entry and coding procedures, although validation studies suggest that variability in coding accuracy or completeness does not have a significant impact on the ability of the eFI to identify changes in frailty status in ageing populations [53–56]. These projections should therefore be considered with some caution, with a focus on patterns and trends rather than specific numbers; further research using eFI2 frailty data would be likely to generate somewhat lower peaks in prevalence. Internal and external validation, however, supported confidence in the simulation projections for health and social care services.

Service use data were not derived from national data. We applied average transition rates by age group derived from longitudinal analysis (MSM) of routine data [6], adjusted for sex, ethnicity, deprivation, urban/rural location and applied average yearly rates of service use by age and frailty group across all simulation years; this approach was supported by internal and external validation. This approach also avoided over-estimating frailty prevalence through use of rates from a population that is more deprived and diverse than average. Application of service use rates by frailty group should also have mitigated this possibility for service use data. Service use was compared with previous analyses where possible, suggesting that the differences in service use noted in more deprived populations are due to higher incidence, in line with other studies [57].

The assumption of average service use rates across all years of the simulation could be erroneous, especially in the context of likely changes to service delivery, funding and access over the period of the simulation, including the pandemic. We have attempted to mitigate this by applying average service use rates per age and frailty group derived from the 8-year data extraction period, use of broad service categories rather than specific services and illustrative scenarios that reflect current policy.

These projections use contacts which do not reflect time, intensity or clinical purpose or meaning. However, these administrative categories map against unit cost categories, which do reflect the different intensity of activity for each event type. In addition, estimates of social care demand will underestimate true demand, because data are only available for publicly funded social care. It is assumed that this will continue to be provided in the same way as it is currently, and that individuals will have to satisfy the same eligibility criteria. It is possible that financial eligibility thresholds will change under social care charging reform and as demand increases [58]. In this case, model projections could over-estimate publicly funded care use. The baseline model also assumes that the same health and social care services will continue to be provided in the same way in the future. Whilst this might not be the case, the scenario experiments reflect likely changes in relation to moving more care into the community, based on current health and social care policy. It should be noted that this analysis used only publicly funded social care contacts. The relatively low number of these contacts should not be interpreted as indicating low demand, which is likely to be substantially higher when privately funded and unpaid care and unmet need, are considered. Evidence suggests that ~37% of care home residents are self-funded [59], as are 23% of domiciliary care users [60], giving some indication of the likely scale of demand.

## Conclusions

Using routine service use data, a simulation model was developed to estimate future demand for community-based services in the ageing population in England. The model forecast increased demand associated with population ageing and increasing frailty. Simulation scenarios of achievable reductions in incidence and progression resulted in modest reductions in demand for these services compared with baseline, but due to large numbers with mild and moderate frailty, demand will continue to increase.

## Supplementary Material

Supplementary_material_afag256

## Research Data Transparency and Availability

The Discover-NOW dataset is coded primary care, secondary, acute, mental health, community health and social care records for over 2.8 million patients who are registered with a GP in NWL. The Discover-NOW dataset is one of Europe’s largest linked longitudinal costed dataset capturing a population of around a third of London in ethnically highly diverse population. This dataset is fed by data from over 400 provider organisations including 344 GP practices. The Discover-NOW dataset is a de-identified dataset used for quality improvement, planning and research, it is controlled by NWL data controllers, and access to it is mediated by the NWL Data Access Committee. The data has been made specifically available for the study and is not otherwise available.
